# *O*-GlcNAc stimulation: A new metabolic approach to treat septic shock

**DOI:** 10.1038/s41598-019-55381-7

**Published:** 2019-12-10

**Authors:** Marine Ferron, Julien Cadiet, Antoine Persello, Valentine Prat, Manon Denis, Angélique Erraud, Virginie Aillerie, Mathieu Mevel, Edith Bigot, John C. Chatham, Chantal Gauthier, Bertrand Rozec, Benjamin Lauzier

**Affiliations:** 1 l’institut du thorax, INSERM, CNRS, UNIV Nantes, Nantes, France; 2 INSERM UMR 1089, Université de Nantes, CHU de Nantes, Nantes, France; 3 Biochemistry Department, Laënnec Hospital, CHU Nantes, Nantes, France; 4 Division of Molecular and Cellular Pathology, Birmingham, United States; 5 l’institut du thorax, INSERM, CNRS, UNIV Nantes, CHU Nantes, Nantes, France

**Keywords:** Cardiac device therapy, Bacterial infection

## Abstract

Septic shock is a systemic inflammation associated with cell metabolism disorders and cardiovascular dysfunction. Increases in *O*-GlcNAcylation have shown beneficial cardiovascular effects in acute pathologies. We used two different rat models to evaluate the beneficial effects of *O*-GlcNAc stimulation at the early phase of septic shock. Rats received lipopolysaccharide (LPS) to induce endotoxemic shock or saline (control) and fluid resuscitation (R) with or without *O*-GlcNAc stimulation (NButGT–10 mg/kg) 1 hour after shock induction. For the second model, rats received cecal ligature and puncture (CLP) surgery and fluid therapy with or without NButGT. Cardiovascular function was evaluated and heart and blood samples were collected and analysed. NButGT treatment efficiently increased total *O*-GlcNAc without modification of HBP enzyme expression.Treatment improved circulating parameters and cardiovascular function in both models, and restored SERCA2a expression levels. NButGT treatment also reduced animal mortality. In this study, we demonstrate that in septic shock *O*-GlcNAc stimulation improves global animal and cardiovascular function outcomes associated with a restoration of SERCA2a levels. This pre-clinical study opens avenues for a potential therapy of early-stage septic shock.

## Introduction

Sepsis is a significant worldwide burden and a leading cause of death. According to Daniels, in 2011 over 20,000 people died worldwide per day from sepsis^1^. A recent consenus report by Singer *et al*. defined septic shock as a systemic inflammatory response syndrome consecutive to an infection associated with both cardiovascular dysfunction and cellular metbolic alterations^2^. Indeed, diagnostic factors of septic shock include hypotension, tachycardia, hyperlactatemia and organ dysfunction.

Myocardial depression occurs in about 50% of septic shock patients^3^. Whilst, the mechanisms involved in myocardial depression are not completely understood, numerous studies have determined that the common features include micro-vascular abnormalities, calcium deregulation, metabolic changes, mitochondrial dysfunction and cardiomyocyte apoptosis^4^. Concomitant inflammatory responses resulting in the synthesis of high cytokine and chemokine levels (e.g. TNFα, IL-6) could also be responsible for myocardial dysfunction after septic or endotoxemic shock.

After antibiotic administration, which is the first recommended line of therapy, the main goal of septic shock treatment is to optimize oxygen supply to tissues to avoid the onset of ischemia and multiple organ failure. Thus, improvements in hemodynamic and cardiovascular function are the first goal for the management of patients^5^.

Much is known about the major metabolic pathways in the heart and the implication of flux alteration during the development of heart failure, however little is known about alternative metabolic pathways. The hexosamine biosynthetic pathway (HBP) leads to a unique form of protein glycosylation that has been studied intensively over the last decade, despite *O*GlcNAcylation being first described in 1984 by Torres and Hart^6^. This posttranslational modification is a rapid and reversible process that regulates the activity or localization of proteins, and *O*GlcNAcylation can compete with phosphorylation on serine and threonine residues. Flux through the HBP is modulated by the rate limiting enzyme Lglutamine fructose-6-phosphate amidotransferase (GFAT) and only 2 enzymes can transfer or remove the GlcNAc moieties on a protein: *O*GlcNAc transferase (OGT) and *O*GlcNAcase (OGA) respectively. The level of *O*-GlcNAc can be modulated using pharmacological compounds to decrease (e.g.: DON and Azaserine) or increase (e.g.: glucosamine, PUGNAc, NButGT) the level of protein *O*-GlcNAcylation. Whilst protein *O*-GlcNAcylation modulation seems to cause different effects in acute and chronic pathologies^7^, there is little evidence to suggest that a global increase in this post-translational modification could become a future therapeutic avenue to treat septic shock. In trauma-haemorrhage situations, glucosamine or PUGNAc administered during reperfusion increase survival of animals through an improvement in inflammatory responses and organ function^8^. More recently, Hwang *et al*. demonstrated in a septic shock model that pre-treatment with glucosamine improves survival thanks to a decrease in the inflammatory state in mice^9^.

In this context, we propose that an increase in protein *O*-GlcNAcylation by NButGT post-treatment could improve the outcome of septic shock in an animal model., We demonstrate that despite no improvement in the inflammatory state, increased protein *O*-GlcNAcylation improves global outcome in our septic shock rats. In our opinion, this treatment resulted in amelioration of cardiac function.

## Results

### NButGT efficiently increases total *O*-GlcNAc in septic shock without any consequence on major enzymes involved in the hexosamine biosynthetic pathway

**Figure 1:**
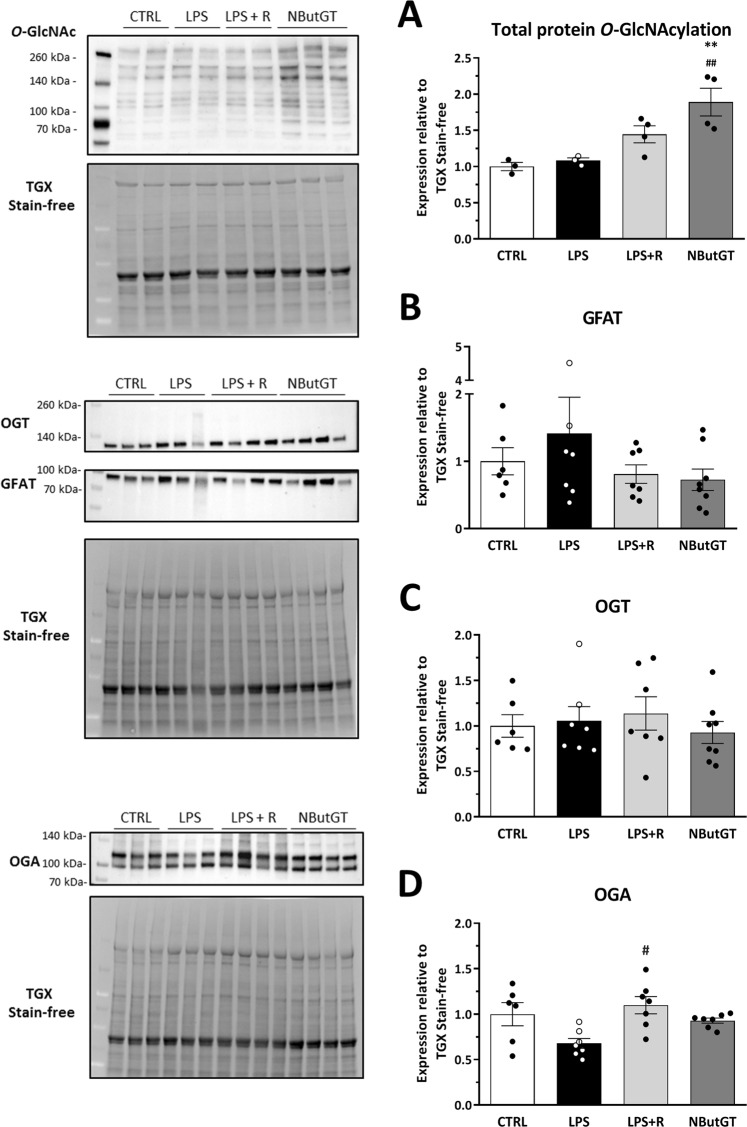
Impact of endotoxemic shock and *O*-GlcNAc stimulation were evaluated by immunoblot analysis for the major proteins involved in the hexosamine biosynthetic pathway. Heart powder protein from control, LPS, LPS+ fluid resuscitation (R: 15 ml/kg 1 hour after shock induction) and LPS+ R + treatment with NButGT (NButGT: 10 mg/kg) 3 hours after shock induction were probed for total *O*-GlcNAc (**A**), GFAT (**B**), OGT (**C**) and OGA (**D**). Images are representative of typical immunoblots on 4–15% TGX Stain-free gels. Results are expressed as the ratio normalized to GAPDH expression. Values are mean ± SEM, **p < 0.01 *vs* control; ^##^p < 0.01 *vs* LPS; determined by Kruskal-Wallis analysis and Dunn’s post-test.

**Figure 2:**
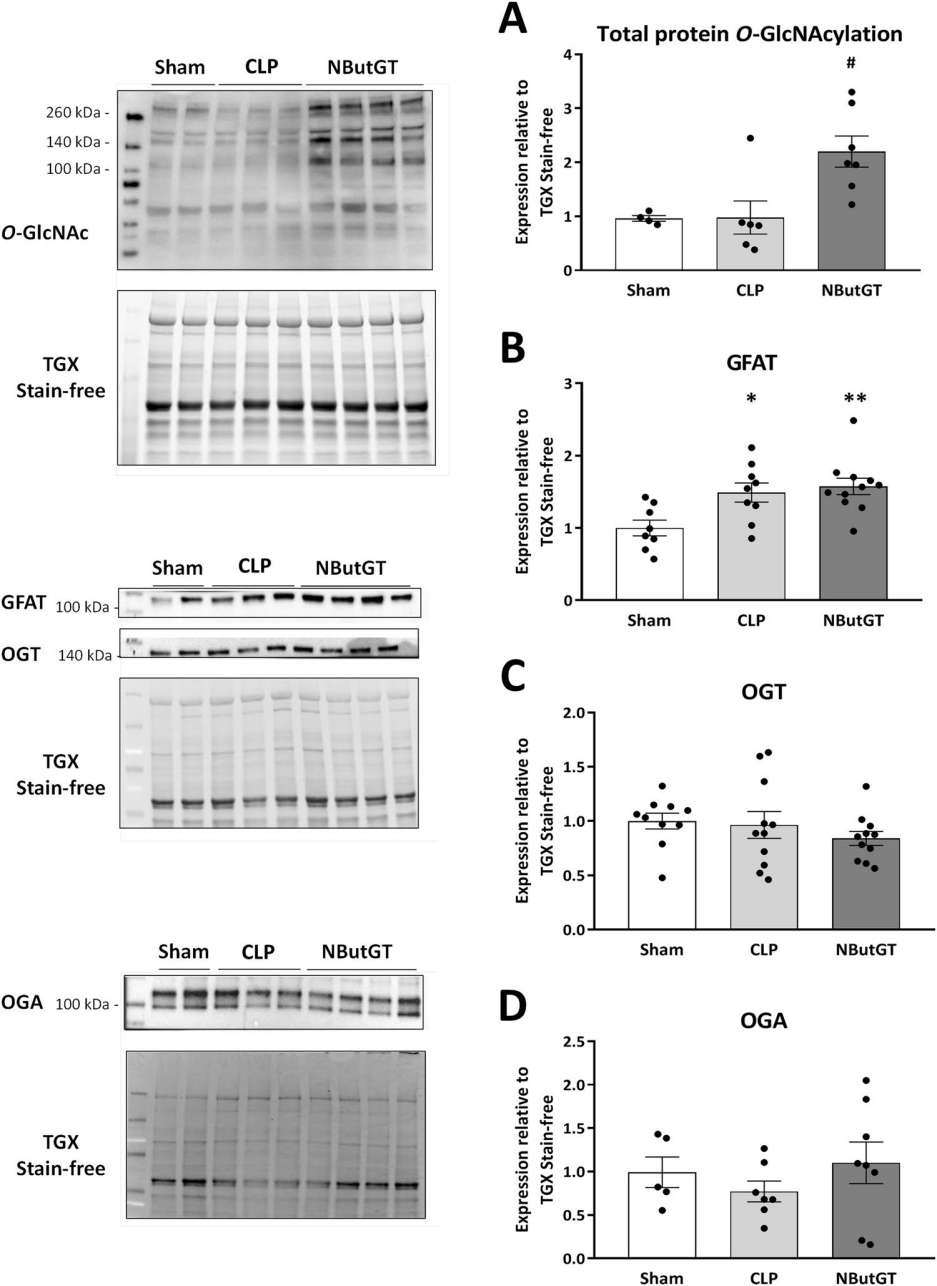
Impact of septic shock and *O*-GlcNAc stimulation were evaluated by immunoblot analysis for the major proteins involved in *O*-GlcNAc pathway regulation. Heart powder protein from Sham, CLP, and CLP + treatment with NButGT (NButGT: 10 mg/kg) 24 hours after surgery were probed for total *O*-GlcNAc (**A**), GFAT (**B**), OGT (**C**) and OGA (**D**). Images are representative of typical immunoblots on 4–15% TGX Stain-free gels. Results are expressed as the ratio normalized to TGX Stain-free or GAPDH expression. Values are mean ± SEM, *p < 0.05, **p < 0.01 vs Sham; ^#^p < 0.05 *vs* CLP; determined by Kruskal-Wallis analysis and Dunn’s post-test.

In septic shock rats (LPS and CLP) the level of *O*-GlcNAcylated proteins and HBP enzymes (OGT and OGA) were not modified when compared to control or Sham rat samples (Fig. 1), but an increase in GFAT, the limiting rate enzyme (p = 0.0210 *vs* Sham Fig. 2B) was observed in the CLP model. Except for OGA, which is slightly increased, neither *O*GlcNAc levels nor OGT and GFAT expression were modified by fluid resuscitation. As expected, NButGT treatment, an OGA blocker, efficiently increased total *O*-GlcNAcylation of proteins 2 fold in the LPS model and by 2.5 fold in the CLP model (Figs. 1A and 2A). In these two models, this increase was not associated with modification of the three main enzymes regulating *O*-GlcNAc levels.

### *O*-GlcNAc stimulation did not impact inflammatory responses

**Figure 3:**
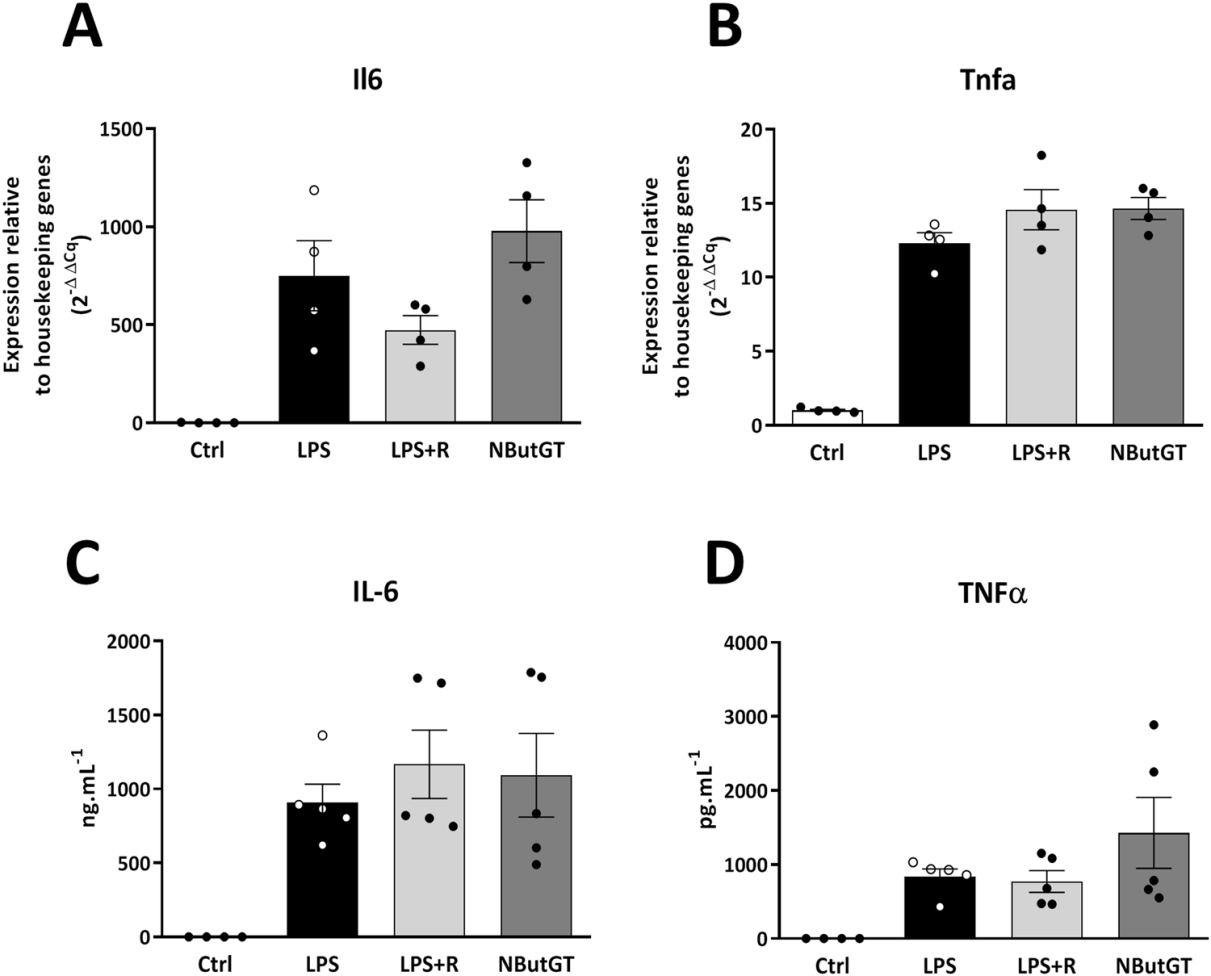
Measurement of inflammatory parameters. Cardiac gene expression and blood cytokine release of Il-6 (**A**) and TNFα (**B**) were evaluated by qRT-PCR and ELISA. Heart powder protein or RNA from control, LPS, LPS+ fluid resuscitation (R: 15 ml/kg 1 hour after shock induction) and LPS+ R + treatment with NButGT (NButGT: 10 mg/kg) 3 hours after shock induction were examined. Values are mean ± SEM, *p < 0.05 *vs* control.

The evaluation of the inflammatory state of septic shock animals was measured on cardiac (RT-qPCR) and plasma (ELISA) samples (Fig. 3). LPS resulted in significant increases in pro-inflammatory IL-6 and TNFα cytokines in both analyses. However, fluid resuscitation alone or completed with NButGT treatment did not modify the inflammatory response of animals.

### *O*-GlcNAc stimulation significantly reduces the impact of septic shock on circulating parameters

**Figure 4:**
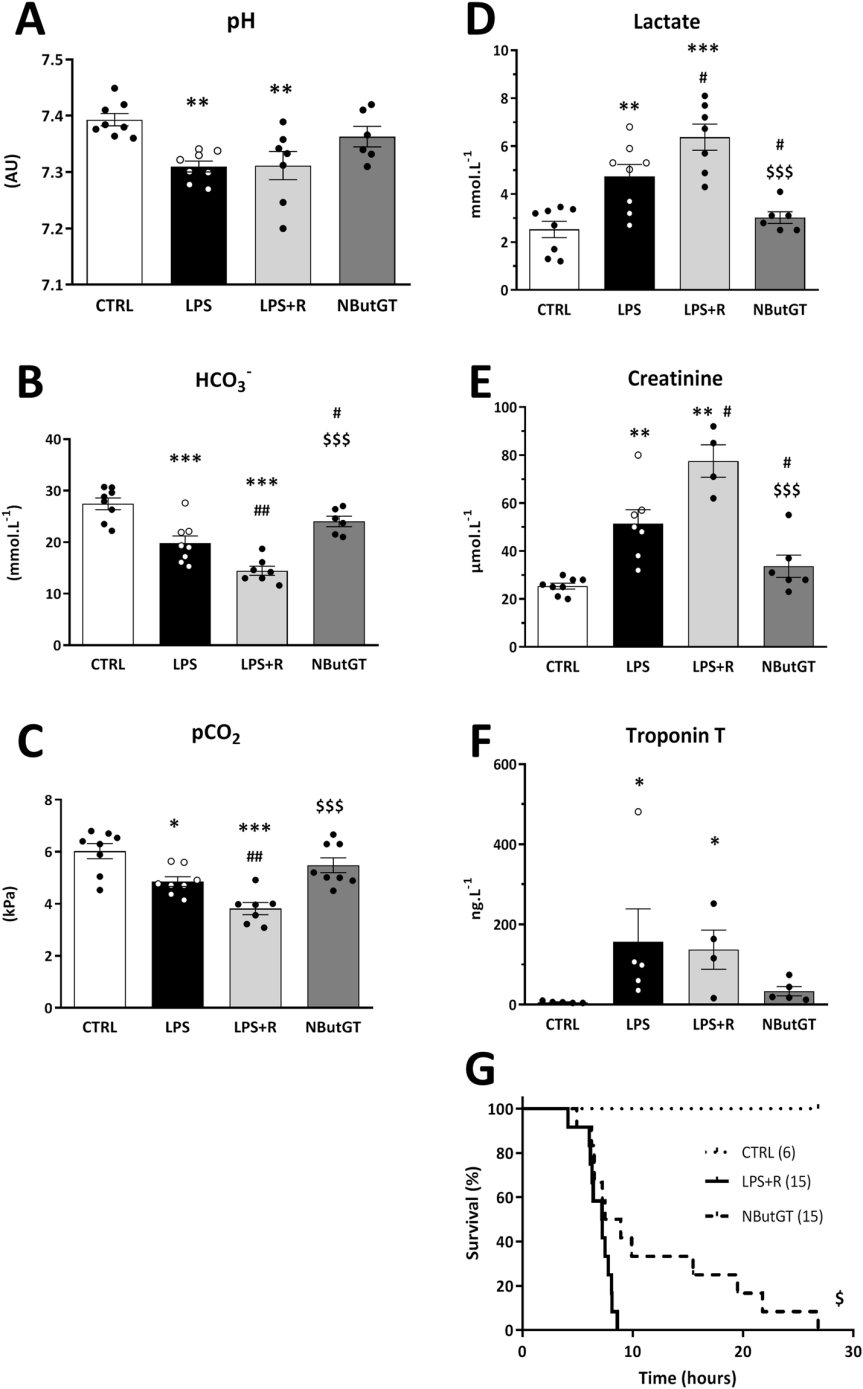
Circulating blood parameters were evaluated 3 hours after shock induction on CTRL, LPS, LPS+ fluid resuscitation (R: 15 ml/kg 1 hour after shock induction) and LPS+ R + treatment with NButGT (NButGT: 10 mg/kg). Parameters representative of homeostasis were evaluated; pH (**A**), bicarbonates (HCO_3_^−^), (**B**) and blood carbon dioxide partial pressure (pCO_2_), (**C**). Lactate was used as a marker of global hypoxia (**D**), creatinine for kidney function evaluation (**D**), and troponin T as a marker of cardiac lesion (**E**). Mortality was evaluated over a 30 hour period following septic shock induction (**F**). Values presented are mean ± SEM. *p < 0.05, **p < 0.01, ***p < 0.001, and were determined by Kruskal-Wallis analysis and Dunn’s post-test. Survival analysis is presented using a Kaplan-Meyer curve and was evaluated using a Mantel-Cox test.

Circulating parameters of the LPS-treated rats showed a reduction in blood pH (pH 7.38 ± 0.02 in control; *vs* 7.31 ± 0.01 in LPS, p < 0.01) associated with a decrease in bicarbonate concentration and carbon dioxide partial pressure (Fig. 4A–C). These rats also presented an increase in circulating markers of hypoxia (lactate, control: 2.52 ± 0.37, LPS: 4.74 ± 0.53 mmol/L, p < 0.01), renal filtration (creatinine, control: 25.3 ± 3.4, LPS: 51.4 ± 7.8 µmol/L, p < 0.01) and cardiac injury (troponin T, CTRL: 6.1 ± 1.0, LPS: 156.5 ± 82.3 ng/L, p < 0.05) (Fig. 4D–F).

While fluid resuscitation alone was not associated with improvement of these parameters, in association with NButGT treatment it normalized circulating parameters such as HCO_3_^−^ (p < 0.001 *vs* LPS + FR) and pCO_2_ (p < 0.001 *vs* LPS + FR). Furthermore, NButGT treatment induced a normalization of lactate (3.02 ± 0.27 mmol/L, p < 0.001 *vs* LPS + R), creatinine (33.7 ± 4.7 µmol/L, p = 0.001 *vs* LPS + R) and troponin T (33.6 ± 11.6 ng/L, ns *vs* LPS + R) indicating improved organ function in NButGT animals. Finally, as shown in Fig. 4G, NButGT supplementation resulted in a 3-fold increase in survival time (26.8 h in NButGT *vs* 8.6 h in LPR + R, p = 0.0242).

### *O*-GlcNAc stimulation improves cardiovascular function in an endotoxemic rat model

**Figure 5:**
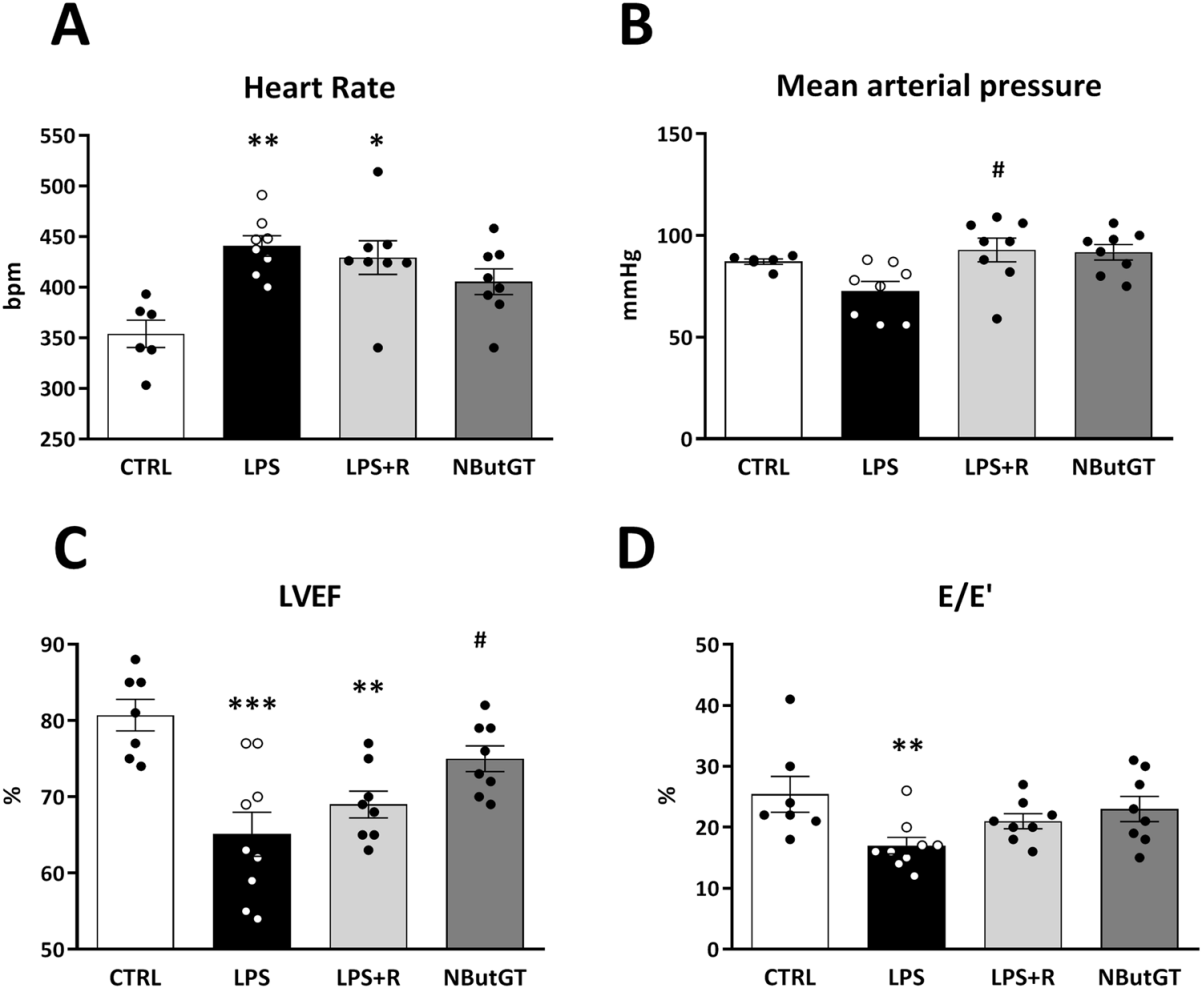
Heart function parameters. Heart function was evaluated *in vivo* 3 hours after shock induction on control, LPS, LPS+ fluid resuscitation (R: 15 ml/kg 1 hour after shock induction) and LPS+ R + treatment with NButGT (10 mg/kg). Upper panel shows results from the invasive evaluation of heart rate (**A**) and mean arterial pressure (**B**). Bottom panel shows various echographic parameters with systolic function evaluation (**C**) left ventricle ejection fraction: LVEF), and diastolic function evaluation (**D**) the ratio between early mitral inflow velocity and mitral annular early diastolic velocity E/E’). Values are mean ± SEM, *p < 0.05, **p < 0.01, ***p < 0.001 *vs* CTRL; ^#^p < 0.05 *vs* LPS; determined by Kruskal-Wallis analysis and Dunn’s post-test.

To explore the putative beneficial effect of *O*-GlcNAc stimulation, our next step was to investigate cardiovascular function. As shown in Fig. 5A,B, endotoxemic shock induced by *iv* injection of LPS lead to hemodynamic alterations especially tachycardia. LPS-treated rats also presented systolic dysfunction (LVEF, 80.7 ± 2.1 in control *vs* 65.1 ± 2.9% in LPS, p < 0.001) and delayed relaxation (E/E′ ratio, 25.4 ± 2.9 in control *vs* 17.0 ± 1.34 in LPS, p < 0.05) evaluated by echocardiography (Fig. 5C,D). Fluid resuscitation efficiently improved mean arterial pressure (similar to the control group) without any impact on heart function.

NButGT supplementation in the fluid resuscitation to stimulate protein *O*-GlcNAcylation did not further impact the mean arterial pressure (similar to the control and LPS + R group). However, it slightly restored cardiac function with an improvement in systolic function (LVEF, 75.0 ± 1.7%, p < 0.05 *vs* LPS) and cardiac relaxation (E/E′, 23.0 ± 2.0, p = 0.090 *vs* LPS).

### Improved plasma parameters and cardiovascular impact of O-GlcNAc stimulation is confirmed in a more relevant model of septic shock

**Figure 6:**
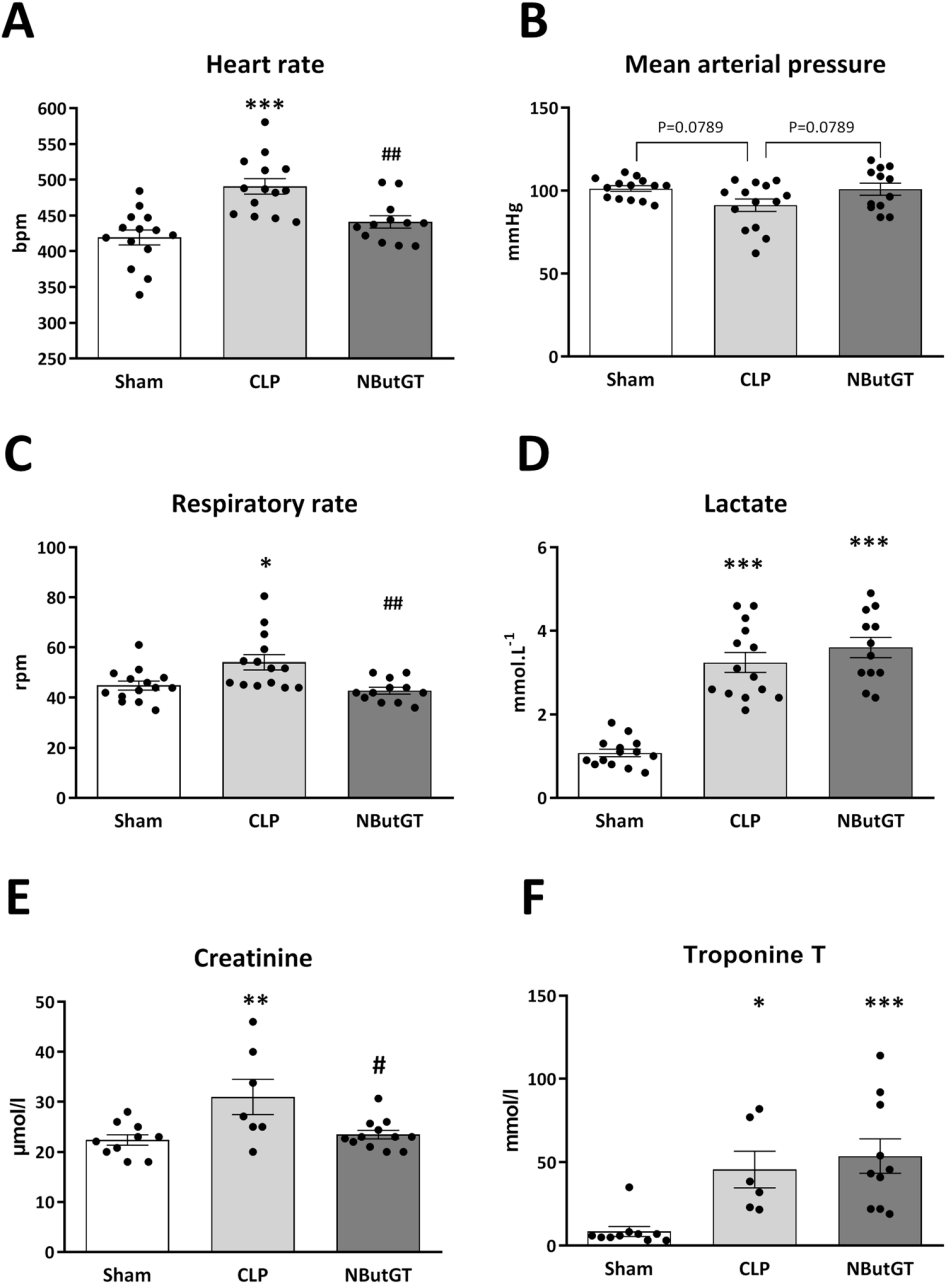
Measures of Global outcome. Global outcome was evaluated *in vivo* 24 hours after surgery on Sham, CLP and CLP+ treatment with NButGT (NButGT: 10 mg/kg). Upper panel presents heart function from the invasive evaluation of (**A**) mean arterial pressure and (**B**) heart rate. Bottom panel represents (**C**) respiratory rate, (**D**) lactatemia (**E**) creatininemia and (**F**) troponin T level. Values are mean ± SEM, *p < 0.05, **p < 0.01, ***p < 0.001 *vs* Sham; ^#^p < 0.05, ^##^p < 0.01 vs CLP; determined using a Kruskal-Wallis test and Dunn’s post-test.

As shown in Fig. 6A,B, CLP-induced septic shock led to hypotension (91.3 ± 3.8 *vs* 101.3 ± 1.7 mmHg in Sham, p = 0.0789) and tachycardia (490.8 ± 10.8 *vs* 419.3 ± 10.6 bpm in Sham, p < 0.001). NButGT supplementation restored mean arterial pressure (101.0 ± 3.6 mmHg, p = 0.0789 *vs* CLP) and reduced heart rate almost to control values (441.1 ± 8.6 bpm, p < 0.01 *vs* CLP).

CLP rats also showed an increase in respiratory rate (54.1 ± 3.0 *vs* 44.9 ± 1.7 rpm in Sham, p < 0.05), in lactatemia (3.2 ± 0.2 vs 1.1 ± 0.1 mmol/L in Sham, p < 0.001 *vs* Sham), and in creatininemia (31.0 ± 3.5 *vs* 22.4 ± 1.0 µmol/L in Sham, p < 0.01). Interestingly, whilst NButGT didn’t improve lactatemia in this model (3.6 ± 0.2 mmol/L), it did significantly improve the respiratory rate (42.8 ± 1.3 rpm, p < 0.01 *vs* CLP) and plasma creatinine levels (23.4 ± 0.9 µmol/L, p < 0.05 *vs* CLP) (Fig. 6C–E).

### *O*-GlcNAc stimulation improves cardiovascular function through restoration of calcium regulators but not changes in autophagy

**Figure 7:**
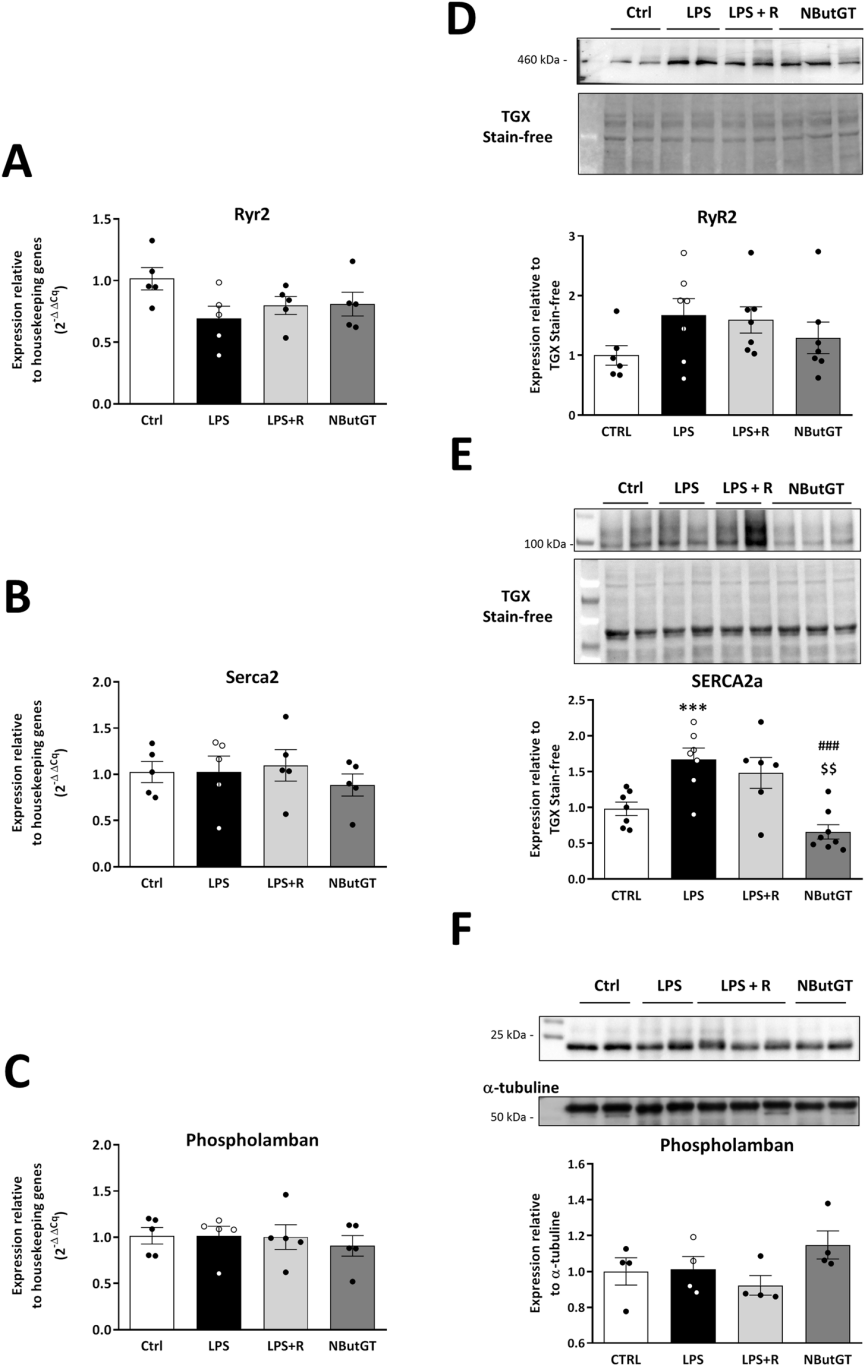
Regulation of Calcium homeostasis. Analysis was performed using heart powder from control, LPS, LPS+ fluid resuscitation (R: 15 ml/kg 1 hour after shock induction) and LPS+ R + treatment with NButGT (NButGT: 10 mg/kg) 3 hours after shock induction. Gene expression of Ryr2 (**A**), Serca2 (**B**) and phospholamban (**C**) were evaluated by qRT-PCR. Protein expression of RyR2 (**D**), SERCA2a (**E**) and phospholamban (**F**) were evaluated by immunoblot analysis. Images are representative of typical immunoblots on 4–15% TGX Stain-free gels. Results are express as the ratio normalized to TGX Stain-free blot or α-tubulin. Values are mean ± SEM, ***p < 0.001 *vs* CTRL; ^###^p < 0.001 *vs* LPS; ^$$^p < 0.01 *vs* LPS+ R; determined by Kruskal-Wallis analysis and Dunn’s post-test.

**Figure 8:**
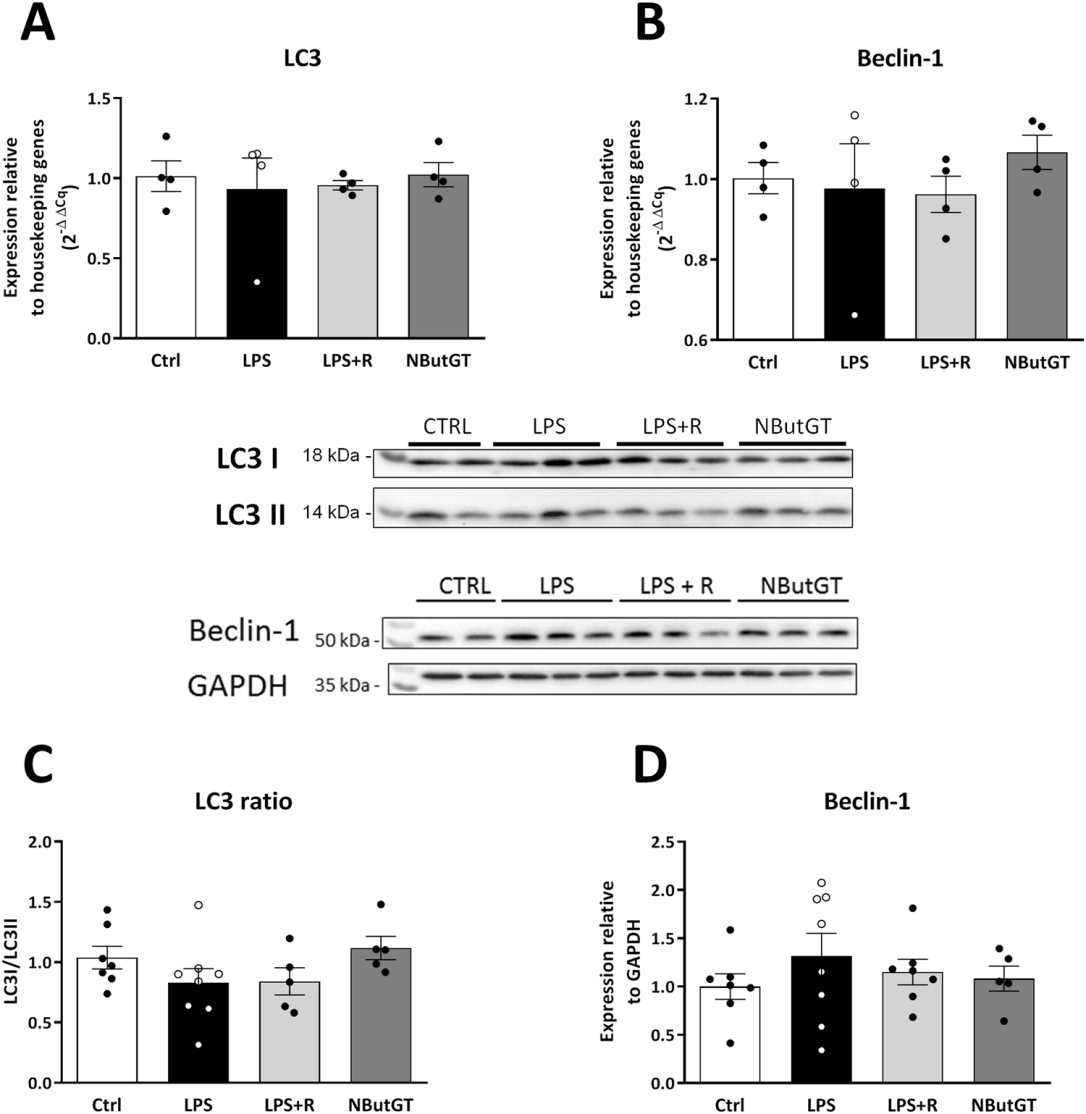
Regulation of Autophagy. Analysis was performed using heart powder from control, LPS, LPS+ fluid resuscitation (R: 15 ml/kg 1 hour after shock induction) and LPS+ R + treatment with NButGT (NButGT: 10 mg/kg) 3 hours after shock induction. Gene and protein expression of LC3 (**A**) and Beclin-1 (**B**) were evaluated by qRT-PCR and immunoblot analysis. Images are representative of typical immunoblots on 4–15% gels. Results are expressed as the ratio normalized to GAPDH expression. Values are mean ± SEM.

The evaluation of the mechanisms involved in the effects of *O*-GlcNAc stimulation in septic shock was only performed for the LPS rat model. In this model, RyR, SERCA and phospholamban gene expression were not modified by LPS, fluid resuscitation nor NButGT (Fig. 7A–C). However, LPS injection did increase SERCA2a protein expression (1.0 ± 0.1 in CTRL *vs* 1.7 ± 0.2 in LPS, p < 0.001), which was not restored by fluid resuscitation (1.5 ± 0.2, p = 0.07 *vs* control) but was impacted by NButGT treatment (0.7 ± 0.1 in NButGT, p < 0.001 *vs* LPS and p < 0.01 *vs* LPS + R) (Fig. 7E). In parallel, neither LPS fluid resuscitation nor NButGT affected RyR2 expression or the active form of phospholamban (Fig. 7D–F). In this model, genes and proteins involved in autophagy were also evaluated in hearts, however these were not modified by NButGT treatment (Fig. 8).

## Discussion

This study was designed to evaluate the therapeutic potential of *O*-GlcNAc stimulation as an early post-treatment for septic shock. Our results demonstrate that an increase in protein *O*-GlcNAcylation with NButGT, an inhibitor of OGA, improved global outcome of animals in two different models of septic shock. *O*-GlcNAc stimulation could be a valuable therapeutic strategy in clinical septic shock management.

In this study, both LPS and CLP models produced a septic shock with organ dysfunction and metabolic perturbations seen at the plasma level (creatinine, troponin T and/or lactate), and cardiovascular dysfunction with pronounced tachycardia, low hypotension, and both systolic and diastolic dysfunction. Thus, according to the latest definition, our animals developed septic shock^2,10^.

Increase in protein *O-*GlcNAcylation with NButGT is not associated with a modification of HBP enzyme expression. Results from our study are based on two models of shock, an LPS-induced model, which develops a rapid and reproducible endotoxemic shock (3 h), and a CLP model which develops a longer term poly-microbial septic shock (24 h). In these two models, septic shock leads to an increase in GFAT expression but did not modify (i) the expression of OGT and OGA, the two direct enzymes involved in *O*-GlcNAc level regulation and (ii) the global protein *O*-GlcNAcylation in cardiac tissue. NButGT administration induced an increase in global protein *O*-GlcNAcylation in heart tissue but had no impact on the expression of OGA, OGT and GFAT. The relationship between *O*-GlcNAc levels and the pathological state is not clearly identified. Whilst our results are in accordance with previous studies demonstrating that intracellular *O*-GlcNAc levels are modulated by stress, regulation of GFAT, OGA and OGT may take a longer. For example, significant increases in intracellular glucose concentrations did not modify GFAT gene or protein expression^11,12^. In parallel, expression of OGT seems to be poorly sensitive to *O*-GlcNAc modulation^13,14^. However, GFAT and OGT activities are regulated by UDP-GlcNAc, GlcN-6-P and *O*-GlcNAc moieties^13,15–17^. *In vitro*, a long term augmentation of *O*-GlcNAc levels with OGT adenovirus transfection or treatment with Thiamet G (10 µM) induced an increase in OGA gene and protein expression^14,15,18^. Collectively, these results suggest that the modulation of OGA requires a longer period of time.

Currently, *iv* fluid resuscitation in association with antibiotic therapy is the gold standard for the treatment of septic shock in patients^19^. As expected, in our study, fluid resuscitation with a bolus of colloid improved mean arterial pressure. However, the circulating markers of acidosis and organ dysfunction were not improved with fluid resuscitation only. Fluid resuscitation therapy has been recently discussed as it may have adverse effects on renal function^20^, with a potential detrimental effect in the long term for patients suffering from septic shock. Results from our study suggest a new interrogation of fluid resuscitation therapy is warranted. Interestingly, the addition of NButGT to the fluid resuscitation treatment in LPS-treated rats efficiently normalized pH and bicarbonates. This therapeutic approach also normalizes circulating markers of organ function such as lactate and troponin T, and improves survival time. Moreover, we observed a significant increase in plasma creatinine levels in LPS-treated rats suggesting acute kidney injury. Treatment with NButGT normalizes this plasma creatinine level in our two models suggesting a potentially beneficial effect on renal function. Additional physiological, biochemical and histological analyses would be necessary to validate this hypothesis. In this model, NButGT also improved cardiac leading to an early improvement in the animal status. The beneficial effects of NButGT were also confirmed in our CLP-induced septic shock model, whereby cardiovascular function and respiration were improved. This observation is in accordance with other studies which also demonstrated the beneficial effects of *O*-GlcNAc stimulation in shock. In cardiac ischemia-reperfusion, after acute renal injury or during haemorrhagic shock, *O*-GlcNAc stimulation (by glucosamine, PUGNAc, siRNA against OGA) improved cardiac or renal function as well as increasing animal survival^8,21–25^. Among the mechanisms underlying these effects, the authors concluded that there were improvements in the inflammatory response, autophagy regulation and/or calcium overload.

Interestingly in our model, *O-*GlcNAc stimulation improves survival, circulating parameters and cardiac function without any impact on the inflammatory response. In our study, IL-6 and TNF-α were elevated 3 hours after shock induction, and treatment with fluid resuscitation with or without NButGT had no impact. Interestingly, acute increase in *O*-GlcNAc level at a later stage of the pathology in other models of heart failure was associated with a significant decrease in inflammatory markers^8^. In a recent study, Baudoin *et al*. defined the relationship between *O*-GlcNAcylation and inflammation as a ‘vast territory to explore’^26^. In fact, the role of *O*GlcNAc on inflammation is controversial. In previous studies of septic shock^9^ or inflammatory models such as in trauma-haemorrhage^27^, the authors demonstrated a reduction in plasma cytokines (IL-6 or TNF-α). Nevertheless, this elevated inflammatory response without improvement attributable to NButGT is concordant with the fact that the pro*-*inflammatory response begins 30 minutes after LPS injection and is maintained for at least 24 hours^28,29^. Other studies have evaluated the impact of increased protein *O*-GlcNAcylation at a later stage and early NButGT treatment might have an effect at a later stage of the pathology.

Autophagy is considered as a biological quality control mechanism, and it could result in massive self-degradation or accumulation of toxic material in septic shock. Reducing autophagy has been shown to improve cardiac contractility^30^. Based on this observation, we hypothesized that improvements observed in NButGT-treated rats could partially be explained by a modulation of autophagy. Indeed, the increase in protein *O*-GlcNAcylation has been shown to blunt the autophagic pathway through a decrease in Beclin-1 or LC3II protein expression^31,32^. However, in our study there was no difference in Beclin-1 or LC3 gene or protein expression in control and LPS-treated rats. According to Sun *et al*., the expression of these autophagic regulators depends on the time and dose of LPS administration^33,34^. Therefore, the effect of NButGT on the autophagic pathway cannot be linked to the beneficial effect of NButGT treatment under our experimental conditions.

Cardiac improvements are also associated with improved calcium regulator expression. Considering the main role of cardiac dysfunction in the septic shock response, we investigated calcium homeostasis, a main controller of cardiac contractility, in the LPS rat model only. Whilst many studies describe an alteration of the calcium cycle at the late stage of septic shock, very few studies are available at the early phase^35^. These studies classically show a decreased or unchanged SERCA2 function during the later phase (6–20 h after endotoxemic shock in mouse and rat models), and Morse *et al*. showed an increase in SERCA2 activity at the recovery phase of septic shock^36^. In our study, SERCA2a was investigated at the early phase (3 h) of septic shock and SERCA2a protein levels appear to be increased. Interestingly, NButGT treatment re-established SERCA2a protein expression. We postulated that in our model, SERCA2a overexpression could be a compensatory mechanism aimed at reducing the potential cytoplasmic calcium overload observed in septic shock. In addition, analysis of calcium function of isolated cardiomyocytes was performed on a limited number of animals (n = 4), and are consistent with results from western blot analysis showing a significant improvement in calcium handling in NButGT treated animal cells (data not shown). Whilst previous studies have demonstrated a potential relationship between modulation of *O*-GlcNAc levels and SERCA2a expression, the exact impact on SERCA2a expression remains unclear. Two studies demonstrated an absence of SERCA2a expression changes following modulation of O-GlcNAc levels^37,38^. However, an increase in O-GlcNAc was also associated with a decrease in SERCA2a mRNA and protein expression in the context of diabetes with a modification of cardiac function^39,40^. Moreover, while SERCA2a can be *O*-GlcNAcylated, the consequence on its activity remains unknown^41,42^. The beneficial cardiovascular effects of NButGT in early septic shock management could be partially due to improved calcium homeostasis protein levels.

In summary, we demonstrate that acute *O*-GlcNAc stimulation by NButGT post-treatment tends to improve cardiovascular function and global outcome in two different models of septic shock. In this study, we show that this improvement is associated with the normalization of proteins involved in the calcium cycle. This pre-clinical study opens new avenues for a potential therapy of acute pathologies and especially septic shock.

## Methods

### Reagents

All reagents and solutions were purchased from Thermo Fisher Scientific Inc. (Waltham, MA) or from Sigma-Aldrich (St. Louis, MO). NButGT was synthesized using MS methods^43^. An NButGT dose of 10 mg/kg was determined from pilot studies as the optimal dose to achieve optimal cardiac protein *O*-GlcNAcylation.

### Animal models

Rats were housed under standard conditions of temperature (21–24 °C), humidity (40–60%) and 12 h light/dark cycle with the light period starting at 07:00. Food and water were available *ad libitum*. Experiments were approved by the ethics committee in charge of animal experimentation, committee of the Pays de la Loire #6, (2687–2015102815445892) and performed in accordance with French law on animal welfare, EU Directive 2010/63/EU for animal experiments, and the National Institutes of Health (NIH) Guide for the Care and Use of Laboratory Animals (NIH Pub. No. 85-23, revised 2011).

### Determination of the optimal dose of NButGT

Determination of the optimal dose of NButGT in healthy rats was based on the study of Macauley and Vocadlo^44^. Three doses were tested in the LPS model and explored at the cardiac level by *O*-GlcNAc measurements. For all subsequent protocols in this study, the dose of 10 mg.kg^−1^ was used.

### Endotoxemic rat model

Endotoxemic shock was induced in ten-week-old male Sprague-Dawley rats (Janvier, Le Genest St, France) by intra-venous injection of lipopolysaccharide (LPS, 5 mg.kg-1, LPS from E. Coli O111:B4, Sigma, France), and was compared to control rats (iv injection of saline). One hour later, LPS-treated rats were resuscitated with Gelofusine (B. Braun) 15 mL/kg alone (LPS+ R) or with NButGT (10 mg/kg). Three hours after LPS injection, rats were investigated for *in vivo* measurements, sacrificed and the left ventricle was collected in liquid nitrogen. Heart powder was obtained by grinding with a mortar.

To evaluate mortality, rats were observed for 30 hours after endotoxemic shock induction. During this period, animals were prematurely euthanized with a lethal dose of pentobarbital (Dolethal^®^, Vetoquinol, Paris, France) if they met specific criteria: incapacity to move, *decubitus* position, difficulty in breathing.

### Cecal ligation and puncture rat model

Cecal ligature and puncture (CLP) surgery was performed on ten-week-old male Wistar rats (*Charles River* Ecully, France) by a modified protocol published by Rittirsch *et al*.^45^. Rats were anaesthetized with a gas-mixture of 1% isoflurane (Forene®, Abbott France, Rungis, France) in O_2_. Two ligations were performed. The first-one was just under the ileo-cecal valve (100% of cecum length) to avoid ligation of the ileo-cecal artery, and so as to in initiate a massive necrosis. The second one was located at the distal part of the cecum (20% of length) and included the artery in order to produce necrosis. Between these two ligations, a single puncture was performed using a 16-G needle. Exactly 0.1 mL of faeces was withdrawn and then distributed in the abdominal cavity. Sham rats were anaesthetized and underwent the laparotomy without cecal ligation and puncture. All animals received buprenorphine treatment (10 µg/kg^−1^, subcutaneously, Buprecare®, Med’Vet, France) before surgery and 8 h post-surgery. CLP rats also received 10 ml/kg^−1^ of saline subcutaneously for fluid resuscitation after surgery, and 8 and 22 h post-CLP, supplemented, or not, with NButGT treatment at 10 mg/kg^−1^. Twenty-four hours after surgery, rats were investigated for *in vivo* pressure measurements, sacrificed, and the heart and blood collected for biochemical analysis.

### *In vivo* cardiovascular evaluation

Three hours after LPS or saline injection, animals were anesthetized with 2% volume of isoflurane and 1 L.min^−1^ O_2_ to limit hemodynamic repercussion. Transthoracic echocardiography was performed using an ultrasound system VIVID7 (*GE Healthcare*, Horton, Norway) equipped with a 10 MHz sectorial probe as previously described^39^. Measurements were made on five cardiac cycles and averaged for each data value.

Pressure measurements were performed through the right carotid artery to evaluate the mean arterial pressure signals and heart rate. The right carotid artery was isolated, ligated at the proximal end and a 2 F microtip pressure catheter was insert (*Millar instruments Inc*, Houston, Texas). Pressure signal and heart rate were recorded using an A/D converter (*EMKA Technologies*, Paris, France) stored and displayed on a computer by the IOX1.5.7 Software System (*EMKA Technologies*). Thereafter, blood and left ventricles were collected for biological and biochemical analysis.

### Blood analyses

Blood gas parameters from arterial samples were analyzed using a Gem Premier 3000 blood gas, Instrumentation Laboratory Werfen, Le Pré Saint Gervais, FRANCE. Lactate was also measured on venous blood samples using the Nova StatStrip^®^ Lactate Point-of-Care, Nova Biomedical, Rungis, France. Serum creatinine was measured by an enzymatic method using creatininase and troponine T Hs measured using immunochemiluminescence on a Cobas 6000 Ce analyzer, Roche, Meylan, France

### qPCR and western blotting analysis

Quantitative RT-PCR was performed as previously described^40^ using SYBR green (StepOnePlus, Thermo Fisher). For each tissue, reverse-transcribed total RNA samples were quantified in triplicate for gene expression. Expression was normalised against the Ywhaz and Gapdh house-keeping genes (from a pool of GeNorm-defined genes) and is expressed using the ddCt method. Primers for the genes measured in this study were: Ywhaz (Fwd AGCGAGGGACATCTGCAAC, Rev CTTTGCTTTCTGGCTGCGAA), Gapdh (Fwd TTGTGCAGTGCCAGCCTC, Rev TGAACTTGCCGTGGGTAGAG), Phospholamban (Fwd GGGTTTGACCAGCAAGCAAG, Rev AGCACAGCTGTCTGGTTTGT), Serca (Fwd GTGGAACCTTTGCCACTCATT, Rev AGCACAAAGGGCCAGGAAAT), Ryr (Fwd TCCTGTGCTGAGGCACTGAT, Rev CTCCATGACCCTCAGGGGA), Lc3 (Fwd GCCCCCACCCCTGAAAGG, Rev AATGACCACAAGATCCACATACCAT), Beclin (Fwd TGGAGTCCCTGACAGACAAATCT, Rev ACCGCAAACCCCAGAA), Il6 (Fwd CCACCAGGAACGAAAGTCAAC, Rev TTGCGGAGAGAAACTTCATAGCT); Tnfa (Fwd TGCCATTTCATACCAGGAGAAAGT, Rev CTTAGGGCAAGGGCTCTTGA).

Western blotting experiments were performed on LV tissue samples as previously described^46^, with extraction in the presence of 10 µM NButGT in T-PER buffer (Thermo Scientific). Proteins were evaluated using the following antibodies: GFAT (Thermo Scientific, 1858781), OGT (Sigma, O-6139), OGA (Abcam, 105217), RyR2 (Abcam, 2868), SERCA2a (Abcam, 2817), Phospholamban (Abcam, 2865), *O*-GlcNAc (Thermo Scientific, 24565), and α-tubulin (Sigma, T9026).

### Data and statistical analysis

Results are expressed as the mean ± SEM of n experiments. If the distribution of the samples respected a Gaussian law, then the comparison of parameters was performed using a one-way ANOVA, otherwise the comparison was performed using a Kruskal-Wallis test. Either test was then analysed using a post hoc Dunn’s test (GraphPad Prism 8). A Pvalue < 0.05 was considered statistically significant. Survival analysis is presented using a Kaplan-Meyer survival curve and was evaluated using a Mantel-Cox test.

## References

[1] Daniels R (2011). Surviving the first hours in sepsis: getting the basics right (an intensivist’s perspective). J. Antimicrob. Chemother..

[2] Singer M (2016). The Third International Consensus Definitions for Sepsis and Septic Shock (Sepsis-3). JAMA.

[3] Parker MM (1984). Profound but reversible myocardial depression in patients with septic shock. Ann. Intern. Med..

[4] Rudiger A, Singer M (2007). Mechanisms of sepsis-induced cardiac dysfunction. Critical Care Medicine.

[5] da Silva Ramos FJ, Azevedo LCP (2010). Hemodynamic and perfusion end points for volemic resuscitation in sepsis. Shock.

[6] Torres CR, Hart GW (1984). Topography and polypeptide distribution of terminal N-acetylglucosamine residues on the surfaces of intact lymphocytes. Evidence for O-linked GlcNAc. J. Biol. Chem..

[7] Ferron M, Denis M, Persello A, Rathagirishnan R, Lauzier B (2018). Protein O-GlcNAcylation in Cardiac Pathologies: Past, Present, Future. Front Endocrinol (Lausanne).

[8] Nöt LG, Brocks CA, Vámhidy L, Marchase RB, Chatham JC (2010). Increased O-linked beta-N-acetylglucosamine levels on proteins improves survival, reduces inflammation and organ damage 24 hours after trauma-hemorrhage in rats. Crit. Care Med..

[9] Hwang J-S (2019). Glucosamine improves survival in a mouse model of sepsis and attenuates sepsis-induced lung injury and inflammation. J. Biol. Chem..

[10] Zaky A, Deem S, Bendjelid K, Treggiari MM (2014). Characterization of cardiac dysfunction in sepsis: an ongoing challenge. Shock.

[11] Paterson AJ, Kudlow JE (1995). Regulation of glutamine:fructose-6-phosphate amidotransferase gene transcription by epidermal growth factor and glucose. Endocrinology.

[12] Robinson KA, Weinstein ML, Lindenmayer GE, Buse MG (1995). Effects of diabetes and hyperglycemia on the hexosamine synthesis pathway in rat muscle and liver. Diabetes.

[13] Yehezkel G, Cohen L, Kliger A, Manor E, Khalaila I (2012). O-linked β-N-acetylglucosaminylation (O-GlcNAcylation) in primary and metastatic colorectal cancer clones and effect of N-acetyl-β-D-glucosaminidase silencing on cell phenotype and transcriptome. J. Biol. Chem..

[14] Zhang Z, Tan EP, VandenHull NJ, Peterson KR, Slawson C (2014). O-GlcNAcase Expression is Sensitive to Changes in O-GlcNAc Homeostasis. Front Endocrinol (Lausanne).

[15] Slawson C (2005). Perturbations in O-linked beta-N-acetylglucosamine protein modification cause severe defects in mitotic progression and cytokinesis. J. Biol. Chem..

[16] Graack HR, Cinque U, Kress H (2001). Functional regulation of glutamine:fructose-6-phosphate aminotransferase 1 (GFAT1) of Drosophila melanogaster in a UDP-N-acetylglucosamine and cAMP-dependent manner. Biochem. J..

[17] DeHaven JE, Robinson KA, Nelson BA, Buse MG (2001). A novel variant of glutamine: fructose-6-phosphate amidotransferase-1 (GFAT1) mRNA is selectively expressed in striated muscle. Diabetes.

[18] Slawson C, Lakshmanan T, Knapp S, Hart GW (2008). A mitotic GlcNAcylation/phosphorylation signaling complex alters the posttranslational state of the cytoskeletal protein vimentin. Mol. Biol. Cell.

[19] Cawcutt KA, Peters SG (2014). Severe sepsis and septic shock: clinical overview and update on management. Mayo Clin. Proc..

[20] Mårtensson J, Bellomo R (2015). Sepsis-Induced Acute Kidney Injury. Crit Care Clin.

[21] Champattanachai V, Marchase RB, Chatham JC (2007). Glucosamine protects neonatal cardiomyocytes from ischemia-reperfusion injury via increased protein-associated O-GlcNAc. Am. J. Physiol., Cell Physiol..

[22] Ngoh GA, Watson LJ, Facundo HT, Jones SP (2011). Augmented O-GlcNAc signaling attenuates oxidative stress and calcium overload in cardiomyocytes. Amino Acids.

[23] Hu J (2017). Augmented O-GlcNAc signaling via glucosamine attenuates oxidative stress and apoptosis following contrast-induced acute kidney injury in rats. Free Radic. Biol. Med..

[24] Suh HN, Lee YJ, Kim MO, Ryu JM, Han HJ (2014). Glucosamine-induced Sp1 O-GlcNAcylation ameliorates hypoxia-induced SGLT dysfunction in primary cultured renal proximal tubule cells. J. Cell. Physiol..

[25] Nöt LG, Marchase RB, Fülöp N, Brocks CA, Chatham JC (2007). Glucosamine administration improves survival rate after severe hemorrhagic shock combined with trauma in rats. Shock.

[26] Baudoin L, Issad T (2014). O-GlcNAcylation and Inflammation: A Vast Territory to Explore. Front Endocrinol (Lausanne).

[27] Nöt LG, Brocks CA, Vámhidy L, Marchase RB, Chatham JC (2010). Increased O-linked β-N-acetylglucosamine levels on proteins improves survival, reduces inflammation and organ damage 24 hours after trauma-hemorrhage in rats. Crit Care Med.

[28] Ertel W, Morrison MH, Ayala A, Chaudry IH (1995). Hypoxemia in the absence of blood loss or significant hypotension causes inflammatory cytokine release. Am. J. Physiol..

[29] Keel Marius, Schregenberger Natina, Steckholzer Ursula, Ungethum Udo, Kenney John, Trentz Otmar, Ertel Wolfgang (1996). Endotoxin Tolerance after Severe Injury and Its Regulatory Mechanisms. The Journal of Trauma: Injury, Infection, and Critical Care.

[30] Turdi S (2012). Cardiac-specific overexpression of catalase attenuates lipopolysaccharide-induced myocardial contractile dysfunction: role of autophagy. Free Radic. Biol. Med..

[31] Marsh SA, Powell PC, Dell’italia LJ, Chatham JC (2013). Cardiac O-GlcNAcylation blunts autophagic signaling in the diabetic heart. Life Sci..

[32] Wani WY (2017). O-GlcNAc regulation of autophagy and α-synuclein homeostasis; implications for Parkinson’s disease. Mol Brain.

[33] Sun Y (2018). Beclin-1-Dependent Autophagy Protects the Heart During Sepsis. Circulation.

[34] Sun, Y., Cai, Y. & Zang, Q. S. Cardiac Autophagy in Sepsis. *Cells* **8** (2019). 10.3390/cells8020141 PMC6406743 30744190

[35] Hobai IA, Edgecomb J, LaBarge K, Colucci WS (2015). Dysregulation of intracellular calcium transporters in animal models of sepsis-induced cardiomyopathy. Shock.

[36] Morse JC (2017). Up-regulation of Intracellular Calcium Handling Underlies the Recovery of Endotoxemic Cardiomyopathy in Mice. Anesthesiology.

[37] Yokoe S (2010). Inhibition of phospholamban phosphorylation by O-GlcNAcylation: implications for diabetic cardiomyopathy. Glycobiology.

[38] Zhu, W., El‐Nachef, D., Yang, X., Ledee, D. & Olson, A. K. O‐GlcNAc Transferase Promotes Compensated Cardiac Function and Protein Kinase A O‐GlcNAcylation During Early and Established Pathological Hypertrophy From Pressure Overload. *J Am Heart Assoc* **8** (2019). 10.1161/JAHA.118.011260 PMC6585351 31131693

[39] Clark RJ (2003). Diabetes and the accompanying hyperglycemia impairs cardiomyocyte calcium cycling through increased nuclear O-GlcNAcylation. J. Biol. Chem..

[40] Hu Y (2005). Adenovirus-mediated overexpression of O-GlcNAcase improves contractile function in the diabetic heart. Circ. Res..

[41] Johnsen VL (2013). Enhanced cardiac protein glycosylation (O-GlcNAc) of selected mitochondrial proteins in rats artificially selected for low running capacity. Physiol. Genomics.

[42] Fricovsky ES (2012). Excess protein O-GlcNAcylation and the progression of diabetic cardiomyopathy. Am. J. Physiol. Regul. Integr. Comp. Physiol..

[43] Macauley MS, Whitworth GE, Debowski AW, Chin D, Vocadlo DJ (2005). O-GlcNAcase uses substrate-assisted catalysis: kinetic analysis and development of highly selective mechanism-inspired inhibitors. J. Biol. Chem..

[44] Macauley MS, Vocadlo DJ (2010). Increasing O-GlcNAc levels: An overview of small-molecule inhibitors of O-GlcNAcase. Biochim. Biophys. Acta.

[45] Rittirsch D, Huber-Lang MS, Flierl MA, Ward PA (2009). Immunodesign of experimental sepsis by cecal ligation and puncture. Nat Protoc.

[46] Merlet N (2013). Increased beta2-adrenoceptors in doxorubicin-induced cardiomyopathy in rat. PLoS ONE.

